# A systematic review on the effect of low-dose radiation on hearing

**DOI:** 10.1007/s00411-021-00926-6

**Published:** 2021-07-24

**Authors:** Srikanth Nayak, Arivudai Nambi, Sathish Kumar, P Hariprakash, Pradeep Yuvaraj, Basavaraj Poojar

**Affiliations:** 1 Department of Audiology and Speech—Language Pathology, Kasturba Medical College, Manipal Academy of Higher Education, Mangalore, 575001 India; 2 Department of Audiology and Speech—Language Pathology, Madras ENT Research Foundation, Chennai, 600028 India; 3 Department of Speech and Hearing, Manipal College of Health Professionals, Manipal Academy of Higher Education, Manipal, Karnataka 576104 India; 4 Department of Speech Pathology and Audiology, National Institute of Mental Health and Neurosciences, Bangalore, Karnataka 560029 India; 5 Department of Pharmacology, Kasturba Medical College, Manipal Academy of Higher Education, Mangalore, 575001 India; 6 Department of Audiology and Speech-Language Pathology, Yenepoya University (Deemed to be University), Mangalore, Karnataka 575018 India

**Keywords:** Low-dose radiation, Ionizing radiation, Hearing loss, Pure tone audiometry

## Abstract

Numerous studies have documented the adverse effects of high-dose radiation on hearing in patients. On the other hand, radiographers are exposed to a low dose of ionizing radiation, and the effect of a low dose of radiation on hearing is quite abstruse. Therefore, the present systematic review aimed to elucidate the effect of low-dose ionizing radiation on hearing. Two authors independently carried out a comprehensive data search in three electronic databases, including PUBMED/MEDLINE, CINAHL, and SCOPUS. Eligible articles were independently assessed for quality by two authors. Cochrane Risk of Bias tool was used assess quality of the included studies. Two articles met the low-dose radiation exposure criteria given by Atomic Energy Regulatory Board (AERB) and National Council on Radiation Protection (NCRP) guidelines. Both studies observed the behavioral symptoms, pure-tone hearing sensitivity at the standard, extended high frequencies, and the middle ear functioning in low-dose radiation-exposed individuals and compared with age and gender-matched controls. One study assessed the cochlear function using transient-evoked otoacoustic emissions (TEOAE). Both studies reported that behavioral symptoms of auditory dysfunction and hearing thresholds at extended high frequencies were higher in radiation-exposed individuals than in the controls. The current systematic review concludes that the low-dose ionizing radiation may affect the hearing adversely. Nevertheless, further studies with robust research design are required to explicate the cause and effect relationship between the occupational low-dose ionizing radiation exposure and hearing.

## Introduction

Radiation has a widespread application in medical practice. Advancements in ionizing radiation technology have revolutionized the diagnostic imaging, radiotherapy, catheters, and other devices used in fluoroscopically guided interventional and nuclear medicine procedures. When ionizing radiation is applied to living tissue, it deposits sufficient energy to produce ions by disintegrating the molecular bonds and dislodge electrons from atoms or molecules. Hence, this electron displacement may affect living cells. Given this ability, ionizing radiation has many clinical applications, such as treating cancer or sterilizing medical equipment. On the other hand, short-term exposure to a high dose of ionizing radiation (2 Gy or more) can cause direct cellular damages, bleedings, coma, or even death within minutes/hours of exposure (Burgio et al. 2018).

In the human body, some systems are more susceptible to radiation-induced adverse effects than other. The magnitude of the adverse effects on cells depends on the cells’ sensitivity to ionizing radiation. Ionizing radiation exposure may cause adverse effects such as cancer, cataract, and congenital anomalies (Bashore 2001; Burgio et al. 2018). The effect of high-dose radiation on hearing is well studied over decades. Numerous studies documented patients who have undergone radiation therapy and developed sensorineural hearing loss (Kwong et al. 1996, Johannesen et al. 2002, Bhandare et al. 2009, Li et al. 2010, Irit Gruss et al. 2012) and conductive hearing loss (Anteunis et al. 1994; Li et al. 2010). A systematic review done by (Raaijmakers and Engelen 2002) states that 1/3 of patients can develop 10 dB or more hearing loss at 4 kHz when a fractionated dose of 2 Gy with a total dose of 70 Gy is applied near the inner ear. Initially, hearing loss was present at high frequencies and progressed towards low frequencies over time (Mujica-Mota et al. 2013). Yang et al.(1997) studied the effect of gamma radiation on the inner ear of guinea pigs that were exposed to a fractionated dose of 2 Gy/day with a total dose of 60 Gy. The auditory effects were visible only after 3 months of radiation exposure. The effects included atrophy of the stria vascularis, degeneration of outer hair cells, and supporting cells of the organ of corti.

Initially, the utility of ionizing radiation was limited to the fields of radiology and radiation oncology. However, with the introduction of catheters, there is an exponential growth in the number of physicians and other specialists utilizing ionizing radiation for diagnosis and treatment (Dotter C.T and Judkins M.P 1964; Navarro et al. 2001; Kim et al. 2008). So professionals who use ionizing radiation will be exposed to low-dose ionizing radiation, and there was a need for damage risk criteria. Hence, various national and international agencies formulated low-dose radiation criteria as less than 20 mSv/year for the safety precautions and should not exceed 100 mSv in 5 years (National Council on Radiation Protection 2001, Atomic Energy Regulatory Board 2018). A handful of studies prove that low-dose radiations below 20 mSv/year still can create long-term health effects such as cancer in humans (Bashore 2001; Nakamura et al. 2013; Burgio et al. 2018). While the effects of low-dose ionizing radiation on cancer susceptibility are determined, its impact on hearing is less researched. Therefore, this study aimed to answer “Does low-dose ionizing radiation affect hearing sensitivity in humans?” A systematic review was carried out in compliance with the preferred reporting items for systematic reviews and meta-analyses (PRISMA) Statement to answer the above question.

## Methodology

### Search strategy

The comprehensive data search was carried out independently by two authors using three electronic databases: PUBMED/MEDLINE, CINAHL, and SCOPUS. The search strategy was based on keywords derived from the PICO approach (participants, intervention/exposure, comparison and outcome). The studies report the outcome of audiological tests (O) in human participants (P) who were exposed to low-dose radiation (I) in comparison to non-exposed group (C) will be considered. The search strings used for the literature search represented in the supplementary material. Further, hand searching was carried out to find relevant articles using back-references of included studies.

### Study selection and data extraction

Articles collected from electronic databases were compiled together in Rayyan QCRI (Ouzzani et al. 2016). Regardless of the study design, all types of studies investigating the effect of low-dose ionizing radiation on the human auditory system were included in the review. In the first step, two independent authors scrutinized the articles and removed the duplicates. Following the duplicate removal, title screening and abstract screening was done based on inclusion and exclusion criteria listed in Table 1. The following data were extracted from the included studies: author and year, study design, participants, exposure duration, outcome measures, results and conclusion. The full length of shortlisted articles was collected for further data extraction process. If there was any disagreement in the selection process at any stage, it was resolved by a third reviewer.

**Table 1:** Inclusion and exclusion criteria for study selection

Inclusion criteria	
1	Participants exposed to radiation below 100 mGy/5 years, or less than 6 mGy/h
2	Articles contain at least one of the standard audiological tests such as pure tone audiometry, immittance audiometry, oto-acoustic emissions, brianstem auditory evoked responses
3	Human studies, regardless of age restriction
4	Articles published till March 2020
5	Articles published in English
Exclusion criteria	
1	Participants with neurological and degenerative disorders or participants with congenital abnormalities
2	Duplicate data published in other included studies

### Quality assessment of included studies

Two independent authors assessed the risk of bias for included studies using the Cochrane ‘Risk of bias’ tool in Revman 5.4.1 (RevMan 2020). The risk of bias was rated as low, high or unclear for the following domains: sequence generation, allocation concealment, blinding, incomplete outcome data, selective outcome reporting and other sources of bias.

The overall certainty of the evidence of each outcome was assessed using the GRADE approach with four possible ratings as high, moderate, low or very low (Ryan and Hill 2016). For the current review, the behavioural symptoms (tinnitus and vertigo), pure tone audiometry and physiological tests (immittance audiometry and TEOAE) were considered as outcome measures. The certainty of evidence states the extent to which the estimated effect was correct. A high certainty of evidence suggests confidence in our estimated effect, and future research is unlikely to modify the certainty of current evidence. The very low certainty of evidence implies that the estimated effect is very uncertain. According to this approach, randomized trials rated as high and other studies rated as low. However, several factors considered for the downgrade of evidence such as study limitations (risk of bias), inconsistency, indirectness of evidence, imprecision, publication bias and as well as upgrade of evidence with respect to the magnitude of effect, dose–response and effect of confounding factors. The change in the level of evidence depends on the seriousness of the factors mentioned above.

## Results

**Fig. 1:**
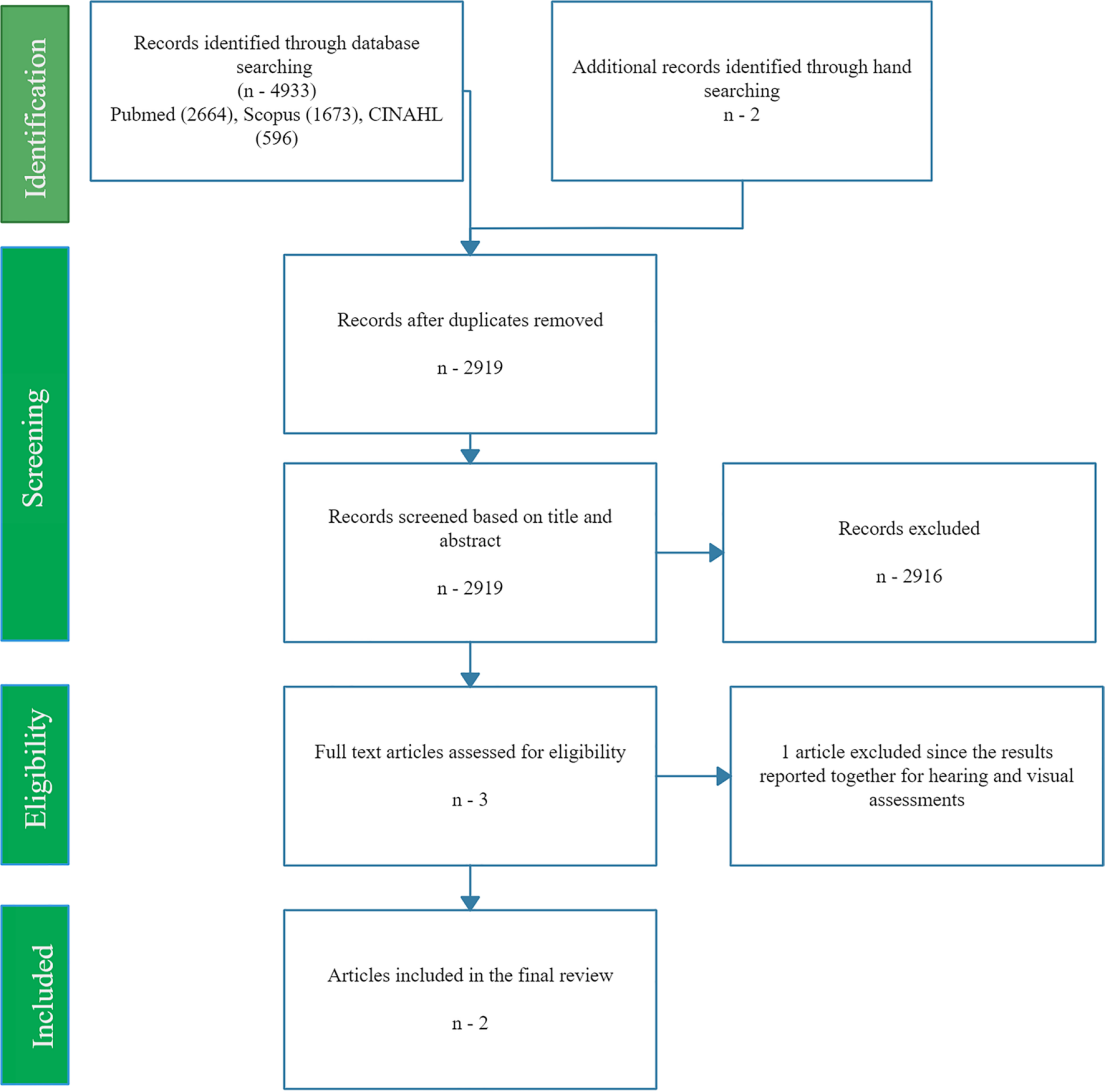
PRISMA chart for the current systematic review

The systematic procedure used for this review is illustrated in the preferred reporting items for systematic reviews and meta-analyses (PRISMA) chart (see Fig. 1). The comprehensive search using keywords yielded 2664 articles in PUBMED, 1673 in Scopus, and 596 articles in CINAHL. Two additional studies were identified by searching bibliographies of included studies. After the removal of duplicates, 2919 articles were obtained. 2916 articles were excluded after title and abstract screening. Only three articles were included for a full-length review. After a full-length article review, one article (Lie et al. 2017) was excluded as the results reported combined for hearing and visual assessment. Hence two articles were included for the current review. In this manner, two articles fulfilled our inclusion criteria and followed data extraction. Table 2 presents the characteristics of the selected articles.

**Table 2:** Characteristics of studies included in the review

Study	Study design	Participants	Exposure duration	Outcome measure	Results	Conclusion
Karlidaǧ et al (2004)	Cross sectional	57 in study group and 32 in age matched control group	4–23 years	PTA, high-frequency audiometry, immittance audiometry (tympanometry and stapedial relflex)	Significant higher hearing threshold found in study group for 4, 6, 8, 14 and 16 kHz compared to the control group. The mean threshold of speech frequencies (500, 1000 and 2000 Hz) for study group were have significant higher threshold compared to control group. No difference found for immittance measurements	Authors suggest that subjects who exposed to radiation for a long period should be evaluated periodically using both standard and high-frequency audiometry could be beneficial
Pooja et al (2018)	Case control	60 subjects in study group and 54 age- and sex-matched subjects in control group	3–19 years	Tuning fork test (Rinne, weber and absolute bone conduction tests), PTA, tympanometry, stapedial reflex and TEOAE at 1, 2 and 4 kHz	Significant correlation found between exposure duration and hearing loss at 500 Hz and 10 kHz. Significant higher hearing threshold found for 12.5 and 16 kHz for study group compared to control group	Most of the frequencies have higher thresholds in the study group compared to controls. But only 12.5 and 16 kHz were significant. Cases also were more symptomatic than controls. If study done using larger group with long radiation exposure duration these changes might become significant

### Subject characteristics and behavioral symptoms of auditory dysfunction

Karlidaǧ et al.(2004) included 57 subjects with a mean age of 29.89 (SD = 6.27) years in the study group. Participants’ work experience ranged from 4 to 23 years (5 h/day). Age- and gender-matched participants with no exposure to radiation were included in the control group. 47 and 24% of the study group individuals experienced tinnitus and vertigo symptoms, respectively. The proportion of individuals who experienced tinnitus and vertigo was significantly higher in the study group than in the control group. Many of their subjects reported that the symptoms appeared after radiation exposure. Pooja et al. (2018) included 60 individuals exposed to ionizing radiation in the study group, plus age and gender-matched individuals with no exposure to radiation in the control group. The participants mean age was 31.32 (SD = 5.64) years, and the work experience ranged from 3 to 19 years. More subjects in the study group reported tinnitus (15%) and vertigo (15%) than the control group. Nevertheless, the difference was not statistically significant.

### Pure tone audiometry

Both studies evaluated behavioral audiometric thresholds using traditional as well as extended high-frequency audiometry. Duration of the radiation exposure had a significant positive correlation with hearing thresholds at 4.8 and 14 kHz (Karlidaǧ et al. 2004) and 0.5 and 10 kHz (Pooja et al. 2018). The mean hearing threshold was higher in the study group than the control group at all frequencies (Karlidaǧ et al. 2004). Similarly, Pooja et al. (2018) also reported a higher hearing threshold in the study group than the control group at all frequencies except 4 and 8 kHz. However, a statistically significant difference was observed at 4, 6, 8, 14, and 16 kHz (Karlidaǧ et al. 2004) and 12.5 and 16 kHz (Pooja et al. 2018).

### Physiological tests

Immittance audiometry and TEOAE were used to assess the middle ear (Karlidaǧ et al. 2004; Pooja et al. 2018) and cochlear function (Pooja et al. 2018), respectively. Both studies did not find a significant difference in static compliance, tympanogram type, middle ear pressure, and acoustic reflexes and were well within the normal range. Pooja et al. (2018) evaluated TEOAE using GSI Audio screener version 3.21. TEOAEs were measured at 2, 3, and 4 kHz, and no statistically significant difference was found between the groups.

### Risk of bias in included studies

**Fig. 2:**
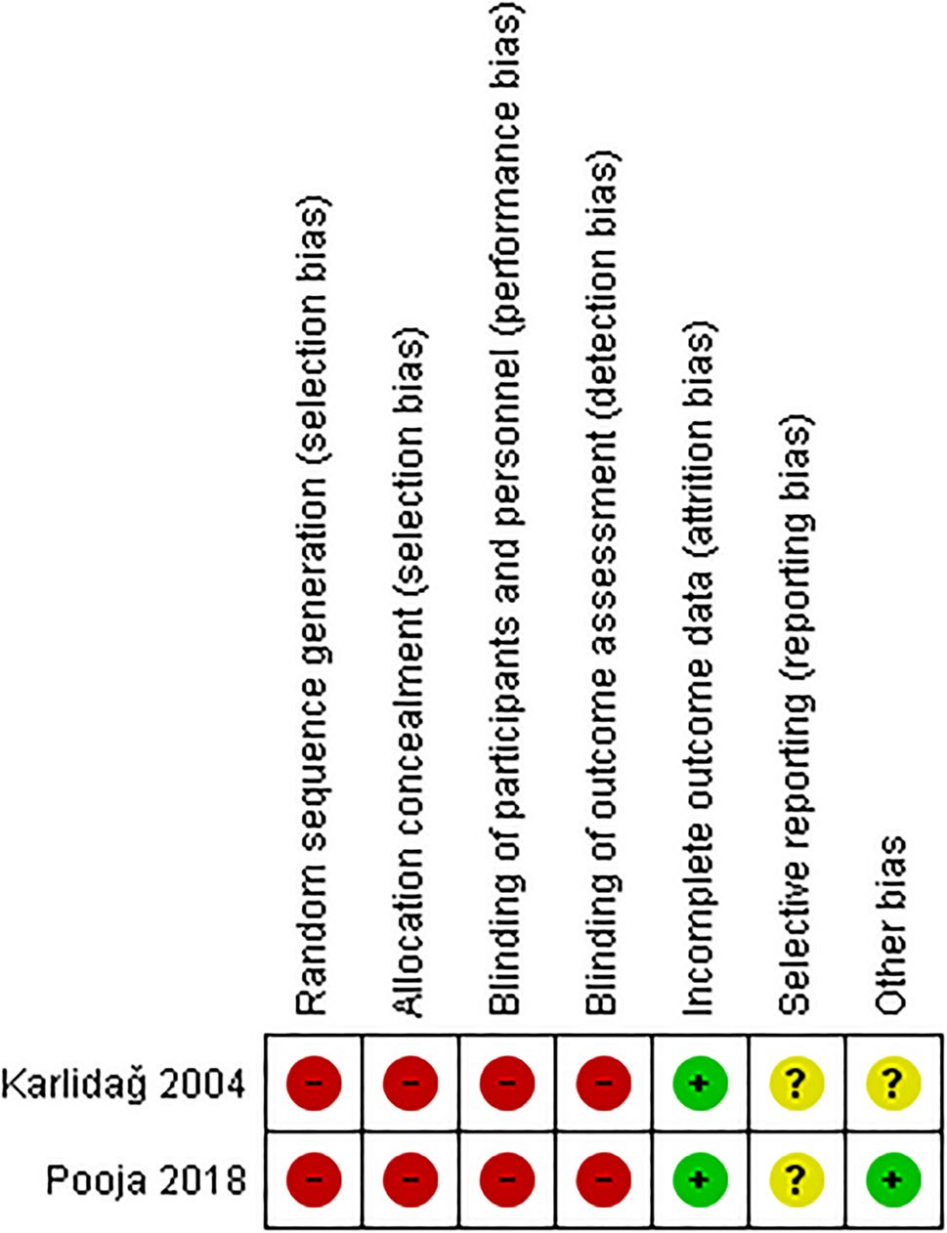
Risk of bias rating by review author’s for each included studies

**Fig. 3:**
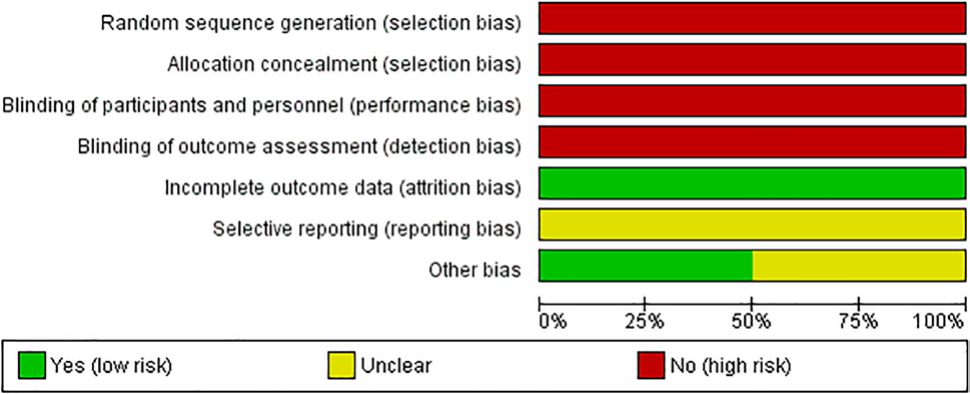
Risk of bias summary for each risk of bias presented in percentages for all included studies

The judgement for risk of bias for each study is presented in Fig. 2, and a summary of those findings in Fig. 3. Since both included studies are observational in nature, no random allocations or allocation concealment would be conducted. Therefore, we rated random sequence generation and allocation concealment as having a high risk of bias. We rated the risk of bias to be high for blinding of participants and personnel, as well as blinding of outcome assessment, since no blinding procedures were reported in any of the included studies. We rated a low risk of bias for incomplete outcome data since no participant dropout information was reported in either of the included studies. We rated the risk of bias for selective reporting as unclear for both studies since no study protocol was available. Pooja et al. (2018) failed to report a detailed description of associated symptoms such as symptom onset durations. Even though no statistical significance obtained for acoustic reflexes and OAE, no descriptive statistics data were reported in the publication. For other sources of bias, we rated the Karlidaǧ et al. (2004) study as an unclear risk of bias because no conflict of interest and funding sources were disclosed in the study. In contrast, Pooja et al. (2018) study rated as having a low risk of bias.

### Certainty of evidence

The certainty of evidence rated as very low for all evaluated outcomes. We downgraded the evidence by two levels due to high risk of bias associated with random assignments of participants and blinding of participants as well as outcome assessments. Further, we downgraded the evidence by one level due to imprecision (small sample size). According to the GRADE approach, very low certainty of evidence states that “very little confidence in the effect estimate. The true effect is likely to be substantially different from the estimate of effect”.

## Discussion

The present systematic review was done to understand the effects of low-dose radiation on human hearing. Two articles met the low-dose radiation exposure criteria given by AERB and NCRP guidelines (National Council on Radiation Protection 2001, Atomic Energy Regulatory Board 2018). Both studies showed that low-dose radiation affects hearing, as evidenced by the higher proportion of individuals experiencing tinnitus and vestibular systems in the study group than the control group, and also a significant difference in the hearing threshold at extended high frequencies. Karlidaǧ et al. (2004) attributed the vascular or cochlear changes due to the radiation as the reason for tinnitus and vertigo, but no possible reasons are discussed in Pooja et al. (2018) study. Though the accurate causal inference is challenging in both studies, the findings should be interpreted cautiously for the reason that both tinnitus and high-frequency hearing loss could be an early sign of progressive hearing loss (Rodríguez Valiente et al. 2016). Electrophysiological evidence and computational models have shown that tinnitus may arise as a perceptual consequence of subtle cochlear dysfunction and hidden hearing loss (Schaette and McAlpine 2011; Gu et al. 2012). Similarly, the presence of the normal hearing threshold for standard audiometric frequencies but elevated hearing threshold at extended high frequencies can also indicate hidden hearing loss (Liberman et al. 2016).

Immittance audiometry revealed normal middle ear function in radiation-exposed individuals in both studies, which disagrees with finding from high-dose radiation studies. Serous otitis media has been commonly observed in individuals with high radiation exposure (Kwong et al. 1996; Johannesen et al. 2002). This result may suggest that the effect of ionizing radiation on middle ear function is dose-specific. Pooja et al. (2018) evaluated cochlear function using TEOAE and found no statistically significant difference between the study and control group. This result suggests that low-dose radiation does not affect the cochlear function significantly. However, the TEOAE may not be an ideal measure to identify the subtle cochlear dysfunction, as it assesses the cochlear functioning only up to 4 kHz. In most cases, the cochlear hearing loss appears in the high frequencies first and then gradually progresses to low frequencies. Therefore, future studies must use high-frequency diagnostic OAEs such as distortion product oto-acoustic emissions (DPOAE) to effectively tap the early cochlear damage (Keefe et al. 2011, 2019).

The current systematic review provides shreds of evidence that low-dose radiation may have a negative impact on hearing. Nevertheless, the precise cause and effect relationship could not be derived based on the findings of both studies. Both studies utilized an observational study design, wherein auditory functions were tested only after occupational radiation exposure. Test findings prior to the occupational radiation exposure, that is, the pre-employment test findings were not reported. Hence, future studies are warranted to investigate the effect of occupational low-dose radiation exposure on hearing with precise control on confounding factors such as premorbid auditory function, age, gender, duration of exposure, and work experience. Future studies should also include tests for hidden hearing loss, electrophysiological measures of neural encoding, and higher auditory processing tests. These tests would provide a better idea about auditory processing difficulties, which may not be evident in conventional audiometric tests.

## Conclusion

The present systematic review aimed to throw some light on the effect of low-dose ionizing radiation on hearing. Two articles met the low-dose radiation exposure criteria given by AERB and NCRP guidelines. Both studies showed that low-dose radiation might have a negative impact on hearing. However, the current review’s evidence shows very low certainty, and we are unable to draw any conclusions about the effect of low-dose radiation on hearing. Further, this review article identified some of the research gaps and future direction for researching occupational low-dose ionizing radiation’s effect on auditory function. Therefore, the current systematic review should stimulate further research in this area.

## Abbreviations

Gy: Gray
mSv: Millisievert
TEOAE: Transient-evoked otoacoustic emissions
AERB: Atomic energy regulatory board
NCRB: National council on radiation protection
